# Therapeutic Targeting of Hepatic Macrophages for the Treatment of Liver Diseases

**DOI:** 10.3389/fimmu.2019.02852

**Published:** 2019-12-03

**Authors:** Daphne van der Heide, Ralf Weiskirchen, Ruchi Bansal

**Affiliations:** ^1^Department of Biomaterials Science and Technology, Faculty of Science and Technology, Technical Medical Center, University of Twente, Enschede, Netherlands; ^2^Institute of Molecular Pathobiochemistry, Experimental Gene Therapy and Clinical Chemistry (IFMPEGKC), RWTH University Hospital Aachen, Aachen, Germany

**Keywords:** hepatic macrophages, targeted therapeutics, liver diseases, monocytes, Kupffer cells

## Abstract

Hepatic macrophages play a central role in maintaining homeostasis in the liver, as well as in the initiation and progression of liver diseases. Hepatic macrophages are mainly derived from resident hepatic macrophages called Kupffer cells or circulating bone marrow-derived monocytes. Kupffer cells are self-renewing and typically non-migrating macrophages in the liver and are stationed in the liver sinusoids in contrast to macrophages originating from circulating monocytes. Kupffer cells regulate liver homeostasis by mediating immunity against non-pathogenic blood-borne molecules, while participating in coordinated immune responses leading to pathogen clearance, leukocyte recruitment and antigen presentation to lymphocytes present in the vasculature. Monocyte-derived macrophages infiltrate into the liver tissue when metabolic or toxic damage instigates and are likely dispensable for replenishing the macrophage population in homeostasis. In recent years, different populations of hepatic macrophages have been identified with distinct phenotypes with discrete functions, far beyond the central dogma of M1 and M2 macrophages. Hepatic macrophages play a central role in the pathogenesis of acute and chronic liver failure, liver fibrosis, non-alcoholic fatty liver disease, alcoholic liver disease, viral hepatitis, and hepatocellular carcinoma, as well as in disease resolution. The understanding of the role of hepatic macrophages in liver diseases provides opportunities for the development of targeted therapeutics for respective malignancies. This review will summarize the current knowledge of the hepatic macrophages, their origin, functions, their critical role in maintaining homeostasis and in the progression or resolution of liver diseases. Furthermore, we will provide a comprehensive overview of the therapeutic targeting strategies against hepatic macrophages developed for the treatment of liver diseases.

## Introduction

The liver is the largest gland in the human body, weighing about 1.5 kg in an adult. It plays an important role in metabolism and mediate several functions including glycogen storage, plasma protein synthesis, and drug detoxification. Liver tissue is highly vascularized with a continuous blood flow and contain extremely permeable fenestrated endothelia facilitating the interaction between the bloodstream and liver cells. The liver is also a central immunological organ in the human body that is exposed to large amounts of circulating antigens and endotoxins from gut microbiota. To maintain liver homeostasis, the liver employs multiple mechanisms to suppress immune responses and create tolerance (1). Liver sinusoidal endothelial cells (LSECs), Kupffer cells (KCs) and dendritic cells are essential players in initiating and shaping liver immune responses, through antigen presentation, and cytokine and chemokine excretion along with neutrophils, B and T lymphocytes, and natural killer (NK) cells that circulate in the hepatic sinusoids (2).

In this review, we present the insights on hepatic macrophages [collectively referred to as resident KCs and monocyte-derived macrophages (MoMFs)], a heterogeneous population of immune cells that originate from different sources (3). Traditionally, macrophages are classified into a classical M1 pro-inflammatory phenotype, and a dichotomic M2 pro-resolving phenotype (3). However, in past years, tremendous heterogeneity in hepatic macrophages, with distinct functions and gene signatures, have been revealed highlighting their cruciality in liver diseases. While KCs represent the main hepatic macrophages during steady state involved in homeostasis, hepatic metabolic or toxic damage results in massive infiltration of MoMFs into the injured liver. Mice with injured livers showed that the infiltration of MoMFs resulted in antigen redistribution between myeloid cell populations and loss of specific markers for tolerogenic phenotype by KCs, due to amplification of the hepatic phagocytic compartment (4, 5). Hepatic macrophages generally maintain homeostasis, but when imbalance ensues this can result in liver inflammation and fibrosis. The imbalance in hepatic macrophage functioning can result in different liver diseases consequently liver inflammation that has been shown to be associated with the poor disease prognosis in patients.

During liver damage, hepatic macrophages interacts and communicates with hepatic stellate cells that are also known as the liver pericytes located in the space of Disse between parenchymal cells and LSECs of the hepatic lobule. Hepatic stellate cells have numerous functions like vitamin A storage, hemodynamic functions, immuno-regulation and extracellular matrix (ECM) remodeling (6). Upon liver injury, these cells transdifferentiate into activated proliferative, migratory and contractile myofibroblasts, and secrete multiple pro-inflammatory and pro-fibrotic factors (7, 8). In addition, these hepatic myofibroblasts promote the differentiation of liver macrophages with pro-inflammatory and pro-fibrotic functions (9), hence play a central role in the development of liver fibrosis and hepatocellular carcinoma (HCC) in combination with liver macrophages.

In the past decades, it has become apparent that macrophages play a central role in initiation, perpetuation and restoration of liver inflammation and damage. Tremendous progress has been made in the understanding of their origin, heterogeneity and functions that has provided with new therapeutics that have been—or currently being—explored in preclinical models and clinical trials. These increasing developments in understanding hepatic macrophage biology will provide new perspectives toward the effective treatment of liver diseases.

## Origin of Hepatic Macrophages

**Figure 1:**
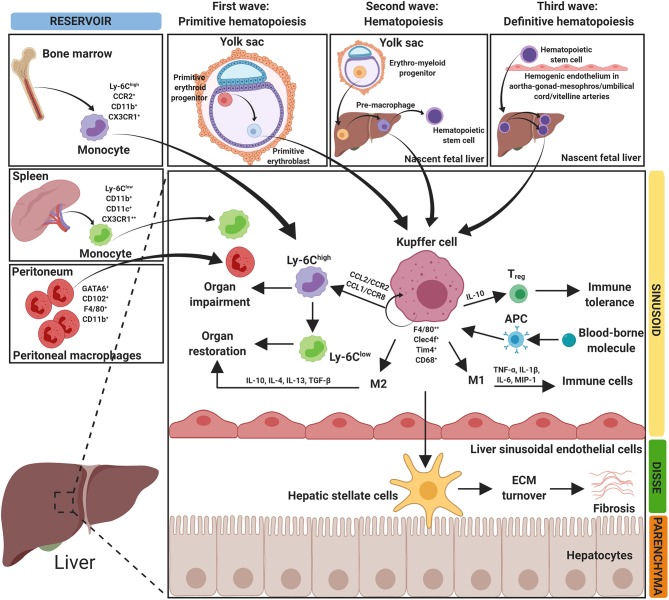
Origin of hepatic macrophages. The figure depicts the diverse origin of hepatic macrophages. Hepatic macrophages can arise from bone marrow derived monocytes, spleen derived monocytes and peritoneum-derived macrophages. Resident hepatic macrophages (Kupffer cells, KCs) develop in three waves at multiple anatomical locations. In the first wave (primitive hematopoiesis), primitive erythroid progenitors originate and differentiate into primitive erythroblasts in the yolk sac. In the second wave (transient hematopoiesis), erythro-myeloid progenitors develop in the yolk sac and migrate into the nascent fetal liver. In the third wave (definitive hematopoiesis), hematopoietic stem cells arise intra-embryonically from hemogenic endothelium in the aorta-gonad-mesonephros region and in the umbilical and vitelline arteries. Hematopoietic stem cells migrate into the fetal liver and expand/differentiate into resident KCs. Hepatic macrophages mediate different functions in organ homeostasis, impairment, restoration, and in fibrosis. APC, antigen presenting cells; CCL, chemokine (C-C) motif ligand; CCR, chemokine (C-C) motif receptor; CD, cluster of differentiation; Clec4f, C-type lectin domain family 4 member F; CX3CR1, C-X3-C motif chemokine receptor 1; ECM, extracellular matrix; F4/80, EGF-like module-containing mucin-like hormone receptor-like 1; GATA6, GATA-binding factor 6; IL, interleukin; MIP-1, macrophage inflammatory protein 1; Tim4, T-cell membrane protein 4; TGFβ, transforming growth factor beta; TNF-α, tumor necrosis factor-α; Treg, regulatory T cells; Ly-6C, lymphocyte antigen 6 complex locus C1.

Ninety percent of all macrophages in the human body reside in the liver. These macrophages can be derived from different cells. The diverse origins of the macrophages result in cellular heterogeneity in the liver, which is reflected in a high diversity in released cytokines, cell surface markers and transcriptional profiles (3, 10). Hepatic macrophages can be derived from either resident hepatic macrophages, called KCs and from distinct populations of infiltrating macrophages i.e., circulating bone marrow (BM)-derived macrophages, avascular peritoneal macrophages (PMs) that reside in subcapsular regions of the liver or splenic monocytes (3, 11–13) (Figure 1). In response to microenvironmental signals, macrophages can migrate and polarize toward different phenotypes with pro-inflammatory and/or anti-inflammatory responses (14).

### Kupffer Cells

KCs are the resident, self-renewing and non-migrating macrophages, localized at the luminal side of the liver sinusoids (3, 10). KCs are the largest tissue-resident macrophage population that maintain liver integrity, restore tissue after injury and infection, and initiate the innate and adaptive immune responses (15). KCs form a highly dynamic and complex network in this defense and mediate tolerance, mostly via the interaction with hepatic regulatory T cells (Tregs) (2). KCs provide an anti-inflammatory microenvironment, during homeostasis, by secreting an anti-inflammatory cytokine interleukin (IL)-10 (4, 12). Antigen presentation to KCs induces CD4 T-cell arrest and secretion of immunosuppressive cytokine IL-10 producing antigen-specific Tregs, which results in promoting the immune tolerance. To maintain liver homeostasis, KCs do not only interact with T cells, but also with another macrophage population, circulating MoMFs and hepatic stellate cells (liver fibroblasts) (Figure 1).

The resident macrophages are developed asynchronously in three waves at multiple anatomical locations (Figure 1). The first wave, primitive hematopoiesis, starts at embryonic day 7.5. In the first wave, primitive erythroid progenitors are detected in the yolk sac (YS) and give rise to primitive erythroblasts (16, 17). In the second wave, transient hematopoiesis, fate-mapping studies in mice showed that resident macrophages descend from a Tie2^+^, also called Tek receptor tyrosine kinase through a cellular pathway generating colony-stimulating factor 1 receptor (CSF1R) positive erythro-myeloid progenitors (EMPs). The EMPs are developed in the YS at embryonic day 8.5, they migrate *via* the bloodstream and colonize to the nascent fetal liver in a chemokine-receptor-dependent manner before embryonic day 10.5 and give rise to the pre-macrophages until embryonic day 16.5. KCs are marginally replaced by hematopoietic stem cells derived macrophages in 1-year-old mice, hereby generating macrophage diversity observed in postnatal tissues (18–20). Finally, the third wave, definitive hematopoiesis, hematopoietic stem cells can be distinguished from other hematopoietic progenitors by their self-renewal capacity, presence in adults and repopulation potential after transplantation (21). Hematopoietic stem cells arise intra-embryonically from hemogenic endothelium in the aorta-gonad-mesonephros region and in the umbilical and vitelline arteries at embryonic days 10.5. The hematopoietic stem cells migrate to the fetal liver, expand and differentiate into resident macrophages (17, 22).

KCs are primarily identified as CD45^+^ F4/80^+^ CD11b^intermediate/int^ cells expressing C-type lectin 4F (*Clec4f*) as a specific KC marker. Based on intravital microscopy, morphometric analysis and gene expression profiling, two distinct intrahepatic KCs subsets i.e., BM-derived KCs and YS-derived KCs, highlighting KCs heterogeneity (23). YS-derived KCs express Macrophage Receptor with Collagenous Structure (MARCO) and T-cell immunoglobulin and mucin domain containing 4 (Tim4) and possess pro-inflammatory functions. On the other hand, BM-derived KCs display an immunoregulatory gene signature possessing protective functions in the maintenance of liver homeostasis (23). Interestingly, recent studies have highlighted a rapid KCs loss upon infection (24, 25), and in HCC (26), thereby questioning why and how KCs loss is established. Several hypotheses have been proposed e.g., KCs loss as a self-inflicted brake of immune system to prevent excessive inflammation, as supported by studies whereby KCs depletion attenuated chronic inflammatory diseases such as non-alcoholic steatohepatitis (NASH) (27, 28). However, in contrast, studies have revealed that tissue-resident macrophages inhibit inflammation after injury and maintain homeostasis (29, 30). With these contradicting findings, it is highly critical to understand if KCs loss is an essential to control liver inflammation or is a consequence of the inflammation. Considering the distinct KC populations (BM-derived and YS-derived) with different functions might explain these contradictions, however further studies are required to deeply understand the underlying mechanisms. These insights will improve our current understanding about liver diseases and will help in developing novel therapeutic strategies targeting specific KC phenotype for the efficient treatment of liver diseases.

### Monocyte-Derived Macrophages

Circulating MoMFs mainly infiltrate into the liver when hepatic metabolic or toxic damage occurs and are likely dispensable for replenishing the macrophage population in homeostasis (Figure 1). MoMFs are mainly derived from a chemokine C-X3-C motif receptor 1 (CX3CR1)^+^ CD117^+^ Lin^−^ (lineage-negative) BM progenitors and express CX3CR1, lymphocyte antigen 6 complex locus C1 (Ly-6C), CD11b and chemokine (C-C motif) receptor 2 (CCR2) (31). MoMFs recruitment is primarily initiated by activated toll-like receptor (TLR) signaling in KCs or hepatic stellate cells that results in increased secretion of chemokine (C-C motif) ligand 2 (CCL2) or monocyte chemoattractant protein 1 (MCP-1) (32–34).

Upon recruitment, MoMFs differentiate into a plethora of phenotypes with discrete functions depending on the microenvironmental cues. Studies in mice have shown that inflammatory lymphocyte antigen 6 complex, locus C1 (Ly-6C)^high^ expressing monocytes (analogous to CD14^hi^ CD16^lo^ human monocytes) are attracted and accumulated in the injured liver tissue depending on chemokine ligand/receptor interactions of CCL2/CCR2 or CCL1/CCR8 (3, 34–37). In acute liver injury mouse model, Ly-6C^high^ monocytes have been shown to express the increasing levels of T cell Ig Mucin 3 (*Havcr2*), toll-like receptors (*Tlr2*), C-type lectins (*Clec4d, Clec4e*, and *Clec5a*), CD209a and CD93 (38). Ly-6C^high^ monocytes provoke organ impairment, but can locally undergo a functional switch into restorative Ly-6C^low^ monocytes (analogous to CD14^low^ CD16^high^ human monocytes) that can restore liver damage. Therefore, this study showed a Ly-6C^high^/Ly-6C^low^ phenotype beyond the traditional M1/M2 classification (39).

Ly-6C^high^ monocytes express high levels of CCR2 and activated hepatic stellate cells have been to secrete increasing levels of CCR2 ligand CCL2. It has been further suggested using CCR2 deficient mouse model, that CCR2 critically controls intrahepatic Ly-6C^high^ monocytes recruitment during liver injury via CCR2-dependent BM egress and promote the progression of liver fibrosis (39, 40). The CCL2/CCR2 signaling therefore critically mediates hepatic macrophage recruitment upon liver injury, leading to shaping of the inflammatory response in the injured liver. Furthermore, other CCRs such as CCR1 and CCR5, via CCL3/MIP1α, CCL4/MIP1β and CCL5/RANTES interaction have been implicated in liver fibrosis attributed to Ly-6C^high^ monocytes recruitment or stellate cells activation, respectively (39–41). CCR8 that interacts with CCL1 was also shown to be involved in the directed infiltration of infiltrating monocytes into injured liver (37). In the respective study, CCR8-deficient mice having reduced intrahepatic monocytes/macrophages showed significantly attenuated hepatic fibrosis that could be restored by adoptive transfer of CCR8-expressing Ly-6C^high^ BM-derived monocytes (37). These studies highlight the importance of MoMFs recruitment in disease progression and therefore the therapeutic potential of CCRs e.g., CCR2, CCR5, and CCR8 antagonists to inhibit MoMFs infiltration thereby limiting liver inflammation and fibrosis.

### Peritoneal and Splenic Macrophages

Wang and Kubes have identified a distinct avascular population of macrophages, PMs, that are recruited through the visceral endothelium upon liver injury and contribute to liver regeneration (13). PMs, that reside in the peritoneal cavity with self-renewal abilities (42), exists as two distinct PM subsets i.e., large peritoneal macrophages (LPMs) and small peritoneal macrophages (SPMs). LPMs originate from embryonic precursors and represent the most abundant subset under steady conditions that display F4/80^high^ CD11b^high^ MHCII^low^ phenotype (Figure 1). While SPMs are the minor subset with F4/80^low^ CD11b^low^ MHCII^high^ phenotype and originate from BM-derived myeloid precursors and predominantly appear during infection (42). Flow cytometry studies confirmed the recruitment of subpopulation of mature PMs expressing CD102 and GATA-binding protein 6 (GATA6) transcription factor within 1 h at the site of thermal injury at the liver surface (13, 43). Furthermore, depletion of PMs prevented the early F4/80^+^ macrophage influx and finally, macrophage recruitment and tissue regeneration was reported to be impaired in GATA6-deficient mice, suggesting an important role of PMs during resolution of liver diseases (13, 43).

Besides KCs, MoMFs and PMs, splenic derived monocytes may also contribute to hepatic macrophages during liver injury (11, 44) (Figure 1). Studies have unraveled spleen as a site for storage and deployment of monocytes, and have identified the contribution of splenic monocytes in regulating immune response during injury (44). Furthermore, splenic macrophages have been shown to promote monocytes infiltration and supports M1 dominant phenotype *via* secretion of CCL2, and regulate KCs activation and hepatic inflammation by releasing of factors such as lipocalin-2 in the portal vein (45, 46). However, more studies are vital to gain insights into distinct phenotypes and functions of splenic macrophages during liver diseases.

## Macrophage Heterogeneity: Beyond M1 and M2 Polarization Dogma

Within hepatic macrophage populations, there is a substantial heterogeneity characterized by a broad spectrum of released cytokines, cell surface markers and transcriptional profiles. Within the simplistic M1/M2 terminology, classically activated M1 macrophages—activated by interferon gamma (IFN-γ) and lipopolysaccharides (LPS)—are pro-inflammatory, microbicidal, tumoricidal, and release numerous inflammatory cytokines e.g., tumor necrosis factor (TNF)-α, IL-1, IL-6, IL-12, IL-15, and IL-18. While alternatively activated M2 macrophages downregulate inflammatory responses and facilitate tissue repair by secreting IL-10, IL-4/IL-13, transforming growth factor (TGF)-β and vascular endothelial growth factor (VEGF)-α. Due to the complex biological characteristics, M2 macrophages can be further sub-categorized into distinct phenotypes based on the stimuli: M2a (induced by IL-4 and IL-13), M2b (elicited by immune complexes), M2c (stimulated by IL-10, TGF-β and glucocorticoids) and M2d (activated by IL-6, TLR ligands and adenosine) (47, 48). M2a macrophages are wound healing macrophages that express high levels of mannose receptor (MR, also called CD206), secrete pro-fibrotic factors such as TGF-β, insulin-like growth factor (IGF), and fibronectin, and contribute to tissue repair. M2b macrophages possess both protective and pathogenic roles, and secrete both pro- and anti-inflammatory cytokines. M2c phenotype display regulatory phenotype, can repress inflammation and fibrosis, and promote tissue repair. In addition, M2c macrophages have the ability to induce regulatory T cells and are involved in the phagocytosis of apoptotic cells. M2d macrophages have phenotypic and functional attributes similar to tumor-associated macrophages (TAMs), and are distinct from M2a-c. M2d constitute the major inflammatory component in tumor, contributing to angiogenesis and metastasis (47, 48).

Strikingly, recent studies have unraveled a complex and spectrum of macrophage polarization states beyond the ancient dogma of M1 and M2 macrophages (11, 49). A recent study, using single-cell RNA sequencing, has provided a comprehensive map of the human liver at a single-cell resolution and revealed distinct intrahepatic monocyte/macrophage populations with unique functional pathways. Furthermore, this study highlighted the disparity between different macrophage populations and biological differences between livers from mice and humans. This recent study describing a transcriptional map of the human liver microenvironment provides a framework for understanding the human liver and the role of different cellular phenotypes that will provide a benchmark for the development of novel immunomodulatory therapies (49).

## Function of Hepatic Macrophages in Liver Diseases

Homeostasis of the liver is important for tolerating and regulating immune responses. Hepatic immune tolerance is mostly dependent on the interaction between KCs and Tregs to create a local suppressive microenvironment, while the recruitment of MoMFs and crosstalk between hepatic stellate cells and macrophages are important determinant for the disease progression/pathogenesis, and tissue regeneration following liver injury. For the development of targeting therapies for liver diseases, especially targeting liver inflammation, it is highly crucial to gain insights into the origin, phenotypic heterogeneity and functions of hepatic macrophages. Here, the role of hepatic macrophages in different liver diseases has been described, including acute liver failure (ALF), liver fibrosis, non-alcoholic fatty liver disease (NAFLD), alcoholic liver disease (ALD), viral hepatitis and HCC.

**Figure 2:**
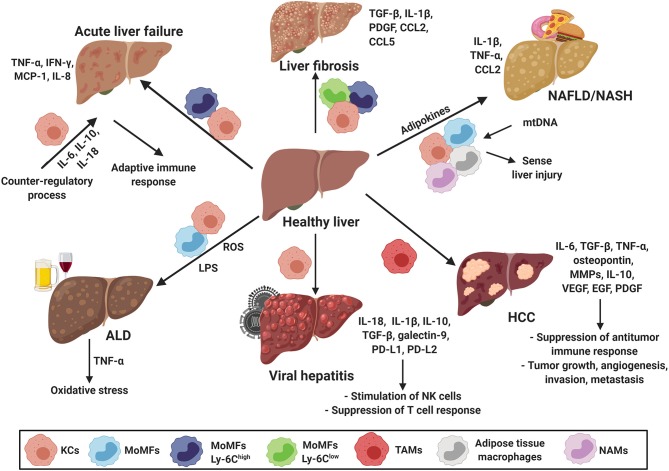
Macrophages in hepatic disease. Hepatic macrophages, KCs and MoMFs, are the key players in various types of liver disease including acute liver failure (ALF), liver fibrosis, non-alcoholic fatty liver disease (NAFLD), alcoholic liver disease (ALD), viral hepatitis and hepatocellular carcinoma (HCC). They modulate adaptive immune responses, fibrosis progression/resolution, sense disease severity, contribute to the establishment of a tumorigenic environment, promote tumor growth, angiogenesis, invasion, and metastasis. CCL, chemokine (C-C) motif ligand; EGF, epidermal growth factor; HCC, hepatocellular carcinoma; IL, interleukin; IFN-γ, interferon-γ; KCs, Kupffer cells; LPS, lipopolysaccharides; Ly-6C, lymphocyte antigen 6 complex locus C1; MCP-1, monocyte chemoattractant protein 1; MMPs, matrix metalloproteinases; MoMFs, monocyte derived macrophages; mtDNA, mitochondrial DNA; NAFLD, non-alcoholic fatty liver disease; NASH, non-alcoholic steatohepatitis; NAMs, NASH-associated macrophages; PD-L, programmed death-ligand; PDGF, platelet-derived growth factor; ROS, reactive oxygen species; TAMs, tumor-associated macrophages; TGFβ, transforming growth factor beta; TNF-α, tumor necrosis factor-α; VEGF, vascular endothelial growth factor.

As mentioned earlier, hepatic macrophages (KCs and MoMFs) are the key players in various types of liver disease. The understanding of their roles in different liver diseases defines them as promising targets to develop new therapies for different liver diseases. Several commendable reviews have detailed the divergent roles and mechanisms of hepatic macrophages in liver diseases (3, 10). The function of hepatic macrophages in these different liver diseases is briefly summarized below and depicted in Figure 2.

### Acute Liver Failure

ALF is a syndrome that is characterized by peripheral vasodilation, encephalopathy and coagulopathy resulting in multiple organ dysfunction and death (50). Patients with ALF, without previously recognized liver disease, sustain a liver injury that results in a rapid loss of hepatic function. Hepatic insult activates KCs and MoMFs to release large amounts of pro-inflammatory cytokines and chemokines, such as TNF-α, IFN-γ, MCP-1/CCL2, and IL-8 (Figure 2). They also express death ligands resulting in hepatocyte apoptosis (50). KCs release wide range of cytokines that are critical in determining the subsequent reactions of other immune cells, hepatocytes and the degree of organ damage (51). MoMFs have been shown to be actively recruited and secrete large amounts of pro-inflammatory cytokines, present antigens *vi*a their surface HLA class II molecules and trigger the adaptive immune response. In ALF, a counter-regulatory process ensues that is intended to offset the damaging effects of unhindered pro-inflammatory KCs and MoMFs activation. This counter-regulatory process consists of KCs secreting cytokines, such as IL-6, IL-10, and IL-18, to compensate for the deleterious effects of the pro-inflammatory response (50, 52).

### Liver Fibrosis

Liver fibrosis is the final common pathway of chronic liver diseases caused by toxic damage, viral infections, autoimmune conditions, and metabolic and genetic diseases (53). The advanced stage of liver fibrosis is called cirrhosis, which is characterized by a loss of architecture, function of the liver and development of life-threatening complications. Hepatocytes, cholangiocytes, hepatic stellate cells, LSECs, immune cells and especially macrophages have been identified in the pathogenesis of liver fibrosis (54). KCs are important in the initial response to injury, produce cytokines and chemokines, and recruit monocytes *via* secretion of CCL2 and CCL5 chemokines (39, 55). Studies have shown that that infiltration of Ly-6C^high^ MoMFs contributing to the expansion of hepatic macrophages to 3–5-fold. MoMFs could promote fibrosis by releasing factors like TGF-β, IL-1β, platelet-derived growth factor (PDGF) and CCL2, which activates hematopoietic stem cells (HSCs) and progresses the inflammation. Ly-6C^high^ MoMFs are pro-fibrogenic and pro-inflammatory (34) and can be switched to Ly-6C^low^ macrophages that are pro-restorative, anti-fibrotic and anti-inflammatory based on microenvironmental cues (39) (Figure 2). Importantly, when the injury in the liver is removed, macrophages are also responsible for the reversal of the liver fibrosis (55).

### Non-alcoholic Fatty Liver Disease

NAFLD refers to a wide range of liver damage, from simple steatosis to NASH, advanced fibrosis, cirrhosis and liver failure (56). Pathological NAFLD resembles alcohol-induced liver disease but occurs in patients who do not abuse alcohol (57). NAFLD is a metabolic syndrome associated with unhealthy lifestyle and several risk factors, obesity, dyslipidemia and insulin resistance (58). Besides liver macrophages, adipose tissue macrophages are also shown to contribute to NAFLD and secretes adipokines and cytokines (59). Hepatic macrophages play the central role in NAFLD, the activation and polarization of macrophages affect the NAFLD progression. Dysregulation in macrophage polarization assist in progression to steatosis, the early stage of NAFLD (60). During chronic liver injury, KCs become activated and release inflammatory cytokines and chemokines. Endotoxins and bacterial components released due to increased intestinal permeability, (lipo)apoptotic hepatocytes, gut microbiota, free fatty acids, cholesterol etc. are the factors that mediate macrophage activation during NAFLD (61). Upon activation, KCs and MoMFs secrete inflammatory cytokines IL-1β, TNF-α and CCL2, that are involved in the development of steatosis, serve as lipogenic factors and promote the inflammatory progression from NAFLD to NASH (62–65) (Figure 2). NASH patients have increased levels of mitochondrial DNA (mtDNA) in serum, as damaged hepatocytes release exosomes/extracellular vesicles containing mitochondrial DNA. KCs recognizes this mitochondrial DNA *via* TLR9 and can therefore sense the severity of the liver injury (66, 67).

More recently, Krenkel et al., has reported the distinct fate of myeloid cells in liver and BM during obesity-related NASH. The study showed a unique, however common and functionally relevant, inflammatory signature in liver myeloid compartment and the BM precursors during NAFLD progression (68). This study suggests that myeloid cells, especially macrophages, adapt their phenotype in response to metabolic microenvironment, indicating the metabolic reprogramming of macrophages in NASH. Interestingly, using single-cell secretome gene analysis, Xiong et al., has identified NASH-associated macrophages (NAMs). NAMs markedly express high levels of triggering receptors expressed on myeloid cells 2 (Trem2) as a feature of mouse and human NASH correlating with disease severity, and responsive to pharmacological and dietary interventions. This study further provide insights into reprogramming of macrophages (and other non-parenchymal cells) in NASH (69).

### Alcoholic Liver Disease

ALD includes different disease stages from simple steatosis to cirrhosis and HCC. Liver injury in ALD can be caused by the following factors: dose, duration and type of alcohol consumption, drinking patterns, sex, ethnicity, obesity, iron overload, viral hepatitis and genetic factors (70). Alcohol ingestion can cause alcohol-induced liver injury through activation of the innate and adaptive immune responses by cytotoxic and reactive oxygen species (ROS)-mediated effects of alcohol and its metabolite, acetaldehyde, on hepatocytes (71). Hepatic macrophages (both M1 and M2 phenotype) increases significantly during disease progression with increased intrahepatic inflammation e.g., increased expression of inflammatory genes, M1 and M2 markers, cytokines and chemokines (70–75).

KCs play a key role in ALD and alcohol consumption can lead to an increase in gut permeability resulting in endotoxemia. KCs can bind to endotoxin *via* CD14 receptor in combination with TLR4, leading to oxidative stress and release of pro-inflammatory cytokines such as TNF-α, causing alcohol-induced liver injury (72–75) (Figure 2). LPS levels are increased in ALD patients, which activates TLR4 signaling in KCs, HSCs and LSECs, and contributes to the regulation of angiogenesis and fibrogenesis, leading to fibrosis. Complement activation, TLR pathways and LPS-mediated pathways, including inflammasome activation could be potential therapeutic targets to develop new therapies for the treatment of ALD (74, 76).

### Viral Hepatitis

Acute viral hepatitis is the most common cause of chronic liver disease worldwide. Chronic hepatitis may progress to cirrhosis, liver failure or HCC (77). A major obstacle in studying viral hepatitis is that there only exist a few immunocompetent animal models for chronic viral hepatitis (10). Hepatic macrophages are recognized as important cells in viral hepatitis. Hepatic macrophages can provide an efficient antiviral response but can also contribute to adverse effects as liver fibrosis or suppression of antiviral immunity (78). KCs play a pivotal role in both the hepatitis B virus (HBV) and the hepatitis C virus (HCV) infection. HBV can infect KCs, leading to the release of inflammatory cytokines, such as IL-18, and stimulation of NK cells (79, 80). The HCV can activate KCs through TLR2, which results in secretion of inflammatory molecules such as IL-1β and IL-18 (81, 82). KCs secrete IL-10, TGF-β, galectin-9 and induces expression of programmed death-ligands 1 and 2 (PD-L1 and PD-L2) during both HBV and HCV infection, resulting in suppression of T cell response (12) (Figure 2).

### Hepatocellular Carcinoma

HCC is one of the most aggressive form of human cancer and a growing cause of cancer-related deaths worldwide (83, 84). Chronic inflammation seems to be essential in the initiation and development of HCC but idem for fibrosis and cirrhosis, which finally result in HCC. HBV and HCV as well as chronic alcohol abuse, biliary disease, metabolic disorders, drugs, toxins and genetic alterations are the major risk factors for the development of HCC (85, 86). TAMs are the key players in cancer-related inflammation (86, 87). TAMs originate from circulating monocytes that are recruited to the tumor microenvironment by CCL2, macrophage colony-stimulating factor (M-CSF), VEGF, and TGF-β, where they differentiate to mature hepatic macrophages (88). In the HCC tumor microenvironment, TAMs are mostly polarized into the M2 phenotype (87, 89) and promote HCC growth, angiogenesis, invasion and metastasis. They are also shown to suppress an antitumor immune response through the interaction with stromal and cancer cells in the tumor microenvironment. In the tumor microenvironment, TAMs release many cytokines, chemokines and growth factors. While IL-6 and TGF-β favor tumor growth, TNF-α, osteopontin, matrix metalloproteases (MMPs), and IL-6 supports invasion and metastasis. TGF-β in combination with IL-10 favors suppression of an antitumor immune response, and VEGF, epidermal growth factor (EGF), PDGF and TGF-β induces angiogenesis (86) (Figure 2).

As described above, hepatic macrophages play an highly essential role in liver diseases like ALF, liver fibrosis, NAFLD, ALD, viral hepatitis, and HCC. Therefore, hepatic macrophages represent the potential targets for therapeutic targeting for the treatment of liver diseases.

## Therapeutic Targeting of Hepatic Macrophages

Based on our increasing understanding about macrophages, several pathways have been identified that regulate their recruitment, differentiation/polarization and activation, based on which, a number of drugs have been designed and investigated in different preclinical murine models. Furthermore, it has been increasingly recognized that macrophages possess high scavenging ability thereby allowing their preferential targeting using nanoparticles (NPs).

However, there are several challenges that are hampering the drug development: (1) disparity in macrophage phenotypes in humans and in animal models resulting in poor translation of therapeutics studied in animal models to human patients; (2) greater macrophage heterogeneity in humans as compared to inbred mouse strains due to several intrinsic (genetics, ethnicity, sex, and age) and extrinsic factors (microbiota, infections, medications); (3) limited in-depth knowledge about human macrophage subsets as compared to mouse models. Importantly, macrophages display incredible heterogeneity with distinct functions in disease initiation and progression as well as protective role and maintain homeostasis. Therefore, it is crucial to target the pathogenic phenotypes of macrophages therapeutically without hindering the functions of so-called restorative or homeostatic macrophages.

**Figure 3:**
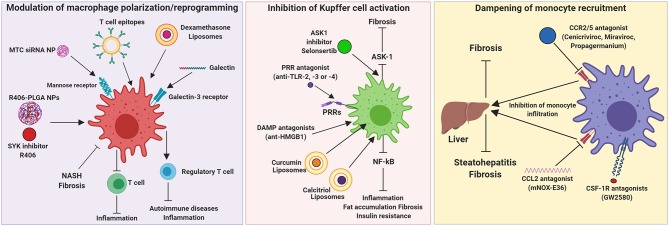
Therapeutic targeting of hepatic macrophages. Modulation of macrophage polarization and function, inhibition of Kupffer cell activation, and dampening of monocyte recruitment into the inflamed liver are the three strategies that have been investigated for the resolution of hepatic inflammation and fibrogenesis. ASK1, Apoptosis signal-regulating kinase 1; CCL, chemokine (C-C) motif ligand; CCR, chemokine (C-C) motif receptor; CSF-1R, colony stimulating factor 1 receptor; DAMP, damage-associated molecular patterns; HMGB1, High mobility group box 1 protein; mNOX-E36, emapticap pegol; MTC, Mannose-modified trimethyl chitosan-cysteine; NASH, non-alcoholic steatohepatitis; NP, nanoparticle; PLGA, poly(lactic-co-glycolic acid); PRR, pattern recognition receptors; TLR, Toll like receptors.

Here, we summarize different approaches that have been explored for targeting of hepatic macrophages and are sub-categorized into three major categories: (a) modulation of macrophage polarization/reprogramming, (b) inhibition of KCs activation and (c) dampening of monocyte recruitment. These strategies have been investigated in experimental animal models, while some have been translated in the clinical settings (Table 1 and Figure 3).

**Table 1:** Therapeutic targeting strategies of hepatic macrophages.

**Strategy**	**(nano) Therapeutics**	**Mechanism/outcome**	**References**
Modulation of macrophage polarization/macrophage reprogramming	Steroids e.g., glucocorticoids	Anti-inflammatory, anti-fibrotic	(90, 91)
	CSF-1R agonists CSF1-Fc	Proliferation of resident macrophages and recruitment of monocytes	(92)
	SLPI	Anti-inflammatory responses through modulation of monocyte/macrophage function	(93)
	MTC-TNF-α siRNA NPs	Inhibition of TNF-α production, reduction in liver inflammation	(94)
	Dexamethasone liposomes	Induction of T cells apoptosis	(95)
	COOH-micelles	Improvement, restoration of tolerance autoimmune disease and chronic inflammation	(96)
	Galectin-3	Inhibits inflammatory macrophage functions	(97)
	SYK pathway inhibitor R406	Anti-inflammatory, anti-fibrotic	(76)
	R406-PLGA NPs	Anti-inflammatory, anti-fibrotic	(98)
Inhibition of KCs activation	ASK1 inhibitor selonsertib	Anti-inflammatory, anti-fibrotic	(99)
	DAMPs (e.g., HMGB1) antagonists	Defected TLR9 signaling, decreased tumor cell proliferationInhibits acute liver injury and bacterial translocation	(90, 100)
	PRR antagonists	Attenuates DAMPs/PAMPs mediated liver injury	(101)
	Curcumin and calcitriol liposomes	Immuno-modulatory	(102)
Dampening of monocyte recruitment	CCR2 antagonists Cenicriviroc, propagermanium	Anti-inflammatory, anti-fibrotic	(103–106)
	CCL2 antagonist mNOX-E36	Anti-inflammatory, anti-fibrotic	(33, 107)
	CCR5 antagonist Miraviroc	Anti-inflammatory, anti-fibrotic	(108)

*ASK-1, apoptosis signal-regulating kinase 1; CCL, chemokine (C-C motif) ligand; CCR, chemokine (C-C) motif receptor; COOH-micelles, carboxy-modified micelles; CSF-1R, colony stimulating factor 1 receptor; CVC, cenicriviroc; KCs, Kupffer cells; DAMPs,/PAMPs damage-associated/pathogen-associated molecular patterns; HMGB1, High mobility group box 1 protein; SLPI, secretory leukocyte protease inhibitor; SYK, spleen tyrosine kinase; mNOX-E36, emapticap pegol; MTC, mannose-modified trimethyl chitosan-cysteine; NPs, nanoparticles; PLGA, poly(lactic-co-glycolic acid); siRNA, small interfering RNA*.

### Modulation of Macrophage Polarization/Reprogramming

Macrophages phenotypes exert contrasting functions, therefore a therapeutic approach that promotes a switch from pathogenic phenotype to restorative phenotype is an interesting approach to accelerate disease resolution and promote liver regeneration. This can be achieved by using therapeutics that promote macrophage polarization and/or using nanoparticles that can selectively reprogram macrophages to restorative phenotype. Steroids (e.g., Dexamethasone), IL-4, IL-10, secretory leukocyte protease inhibitor (SLPI), prostaglandin E2 (PGE2) and colony-stimulating factor 1 receptor (CSF-1R) agonists are the promising therapeutics targeting different immuno-modulatory pathways that have been explored for macrophage reprogramming in liver diseases (90).

NPs are materials with dimensions between 10^−9^ and 10^−7^ m. Such particles can be used as nanocarriers for diagnosis and targeted delivery of therapeutic agents for liver diseases (109). There are several nanocarriers that can be used for diagnosis and therapy referred to as nano-theranostics. The NPs for drug and gene delivery systems include polymeric NPs, lipid NPs, organic and inorganic NPs (110). NPs provide multiple new properties, which can be exploited to improve the ability to detect, treat, monitor and prevent diseases. Moreover, the interactions between these nanomaterials and comparably sized physiological structures in the human body e.g., DNA, proteins and organelles, can be used in combination with existing medical diagnostic and treatment strategies to develop more efficacious approaches (111). These NPs could be loaded with small drug molecules, proteins, DNA or RNA. The drug release can be favorably tuned in different NPs, and in some cases, the release of the therapeutic agents could also be initiated by internal stimuli such as pH (112), or external stimuli such as light (113). Various surface modifications can be applied to NPs including small drug molecules, antibodies, fluorescent dyes, peptides, proteins, polyethylene glycol (PEG), DNA or RNA.

The NPs can be delivered at the targeted site *via* active or passive targeting. With active targeting, the NP surface can be modified with targeting ligands, e.g., targeting peptides, antibodies, which leads to specific binding to the targeted cells. Unlike active targeting, passive targeting does not use any targeting ligands, but uses the physiological properties e.g., enhanced permeability and retention (EPR) in tumors, due to leaky vasculature, to deliver the NPs to the target cells (114). To prolong the circulation time of the NPs in the circulatory system, with both active and passive targeting, the NPs can be surface modified with PEG.

NPs can be made out of different nanomaterials and are generally non-toxic. Gold NPs (AuNPs), which are similar to other inorganic NPs, are mostly non-toxic, but when used in small sizes of 1.4 nm they showed an increased toxicity. Silica NPs induces toxicity due to activation of macrophages. However, at higher doses, many nanotherapeutics have been shown to be toxic. To reduce non-specific uptake by macrophages and alter the response of immune cells, NPs can be modified with PEG or with peptides (115). Liver inflammation and fibrosis can be targeted by nanomedicine. This could be done by the therapeutic targeting of macrophages and especially KCs, because these macrophages have an inherent ability of efficient and non-specific uptake of most nanomaterials and these macrophages play a critical function during inflammation and fibrogenesis. KCs can be targeted by the mannose receptor in liver disease or become activated by specific nanomaterials like peptide-modified gold nanorods (AuNRs) to polarize them into the pro-inflammatory phenotype (115).

Different strategies have been proposed for hepatic macrophage targeting with nanomedicine in the preclinical setting (Table 1 and Figure 3). A system studied by He et al., was to inhibit TNF-α using small interfering RNA (siRNA) delivered *via* mannose-modified trimethyl chitosan-cysteine (MTC) conjugated NPs that are mostly internalized by macrophages, due to macrophages-specific delivery route using the mannose receptor. With this strategy, inflammation-driven liver damage and lethality induced by acute lipopolysaccharide/D-galactosamine administration *in vivo* in mice was prevented. This system offers possibility for oral delivery, which is advantageous for clinical application (94).

Bartneck and colleagues showed that dexamethasone-loaded liposomes were efficient *in vivo* by ameliorating inflammatory liver diseases in a model of acute hepatitis and in chronic carbon tetrachloride (CCl_4_)-based chronic toxic liver injury. This approach resulted in a M2 activation profile of macrophages and with a significant reduction in the number of T cells in the liver (95). Herkel and co-workers published a patent based on a study of the induction of tolerance in liver by influencing Tregs by LSEC- and KC-directed carboxy-modified micelles. The micelles have been modified with T cell epitopes on their surface and were targeted to LSECs and KCs. These micelles can deliver antigens and induce the generation of Tregs to suppress autoimmunity. These NPs are intended to induce tolerance against autoantigens and therefore ameliorating autoimmune diseases and chronic inflammation (96). Traber and Zomer used galactin-3 inhibitors to target inflammatory macrophage functions in liver diseases in mice. The treatment with galectin-3 resulted in regression of NASH and fibrosis. Therefore, they suggested that this galectin-targeting drugs have potential in treatment in human for NASH and fibrosis (97).

Furthermore, Bukong et al., demonstrated the central role of spleen tyrosine kinase (SYK) in multiple proinflammatory pathways involved in the ALD pathogenesis. Furthermore, SYK inhibitor R406 abrogated immune cell infiltration, macrophage and inflammasome activation, thereby ameliorated liver injury, liver inflammation, and reduced hepatic steatosis induced by alcohol (76). In another recent study, Kurniawan and colleagues reported an efficient delivery of small molecule SYK kinase inhibitor R406 using Poly(lactic-co-glycolic acid) (PLGA) NPs for the treatment of NASH in Methionine-Choline-deficient (MCD)-diet induced NASH mouse model (98).

### Inhibition of Kupffer Cell Activation

When liver injury ensues, KCs initiate inflammatory cascades in the liver *via* different mechanisms. For instance, the early communication of cellular distress or hepatocyte damage is mediated by KCs through damage-associated molecular patterns (DAMPs)/pathogen-associated molecular patterns (PAMPs) via pattern recognition receptors (PRRs) and NF-κB signaling and inflammasome activation etc. Therefore, inhibition of PRRs using TLR2, TLR3, and TLR4 antagonists have been shown to ameliorate liver inflammation in murine models (101). Another interesting strategy that has been explored is targeting of released DAMPs such as high mobility group box 1 (HMGB1) proteins and histones. Intriguingly, HMGB1 neutralizing antibodies are shown to attenuate liver injury and reduce bacterial translocation *in vivo* in murine models (90). A possible targeting strategy to treat liver diseases is to influence KCs activation. There are several approaches for influencing KCs activation, such as reducing bacterial translocation and inhibition of TLR4-dependent macrophage activation using a broad spectrum of antibiotics. This could improve steatohepatitis, liver fibrosis and HCC (10).

Modifying KCs activation has been explored (Table 1 and Figure 3). Hepatic macrophages and hepatocytes share some of the intracellular inflammatory signaling pathways like NF-κB, ASK1, JNK, or p38 (116). Loomba et al., developed selonsertib, an inhibitor of the inflammatory signaling pathway ASK1 (apoptosis signal-regulating kinase 1). Selonsertib treatment has been shown to have an effect on hepatocyte metabolism as well as macrophage activation. In a randomized phase 2 trial, selonsertib showed an improvement in fibrosis, lobular inflammation and serum biomarkers of apoptosis and necrosis in patients with NASH and fibrosis (99). However, in phase 3 randomized double-blind placebo-controlled STELLAR-3 and STELLAR-4 studies in patients with F3 fibrosis and compensated F4 cirrhosis respectively, due to NASH, selonsertib did not show a histologic improvement in fibrosis however well-tolerated safety results. In the study of Maradana et al., liposome-encapsulated lipophilic curcumin or 1,25-dihydroxy-vitamin D3, also called calcitriol, was investigated in mice with diet-induced NASH. Curcumin and calcitriol are both NF-κB inhibitors and were shown to be taken up by hepatic macrophages and dendritic cells, leading to the suppression of hepatic inflammation, fat accumulation, fibrosis and insulin resistance (102).

### Dampening of Monocyte Recruitment

MoMFs are recruited by KCs to the liver, where they amplify and maintain liver inflammation. The recruitment of monocytes by KCs is driven by chemokine receptor interactions of CCL2/CCR2 or CCL1/CCR8 (16–19). A strategy to reduce the number of monocytes recruited into the liver include an interference with chemokine signaling. Interference with chemokine signaling can be achieved with monoclonal antibodies against chemokines or receptors, receptor antagonists, inhibition of chemokines by aptamer molecules or small molecule inhibitors blocking chemokine-induced intracellular signaling (10).

Chemokine interference as a targeting therapy for treating liver diseases has been intensively studied (Table 1 and Figure 3). Lefebvre and co-workers as well as Krenkel and colleagues showed that the dual CCR2/CCR5 inhibitor, called cenicriviroc (CVC) that efficiently blocks CCL2-mediated monocyte recruitment to the liver and has an anti-fibrotic effect in mouse models of liver and kidney fibrosis (103, 104). In a randomized controlled trial, Friedman et al., showed ≥2-point improvement in NAS with no worsening of fibrosis after 1 year of CVC treatment in patients with NASH (NAS ≥ 4) and stage 1–3 liver fibrosis (105). Baeck et al., investigated CCL2 inhibitor, RNA-aptamer molecule mNOX-E36 in CCl_4_ fibrosis model and MCD-diet induced NASH model. mNOX-E36 inhibited early influx of Ly6C^+^ monocytes thereby shifting macrophage equilibrium to Ly6C^−^-restorative monocytes hence favoring fibrosis resolution (33, 107). Furthermore, Mulder et al., showed that CCR2 has a crucial role in the recruitment of immune cells to white adipose tissue and the liver, and a CCR2 inhibitor, propagermanium, attenuated liver inflammation and NASH development (106). Interestingly, chemokine CCL5/RANTES has been documented to play an important role in the progression of hepatic inflammation and fibrosis. Maraviroc, a CCL5/RANTES inhibitor, ameliorated hepatic steatosis in a high-fat diet (HFD)-induced model of NAFLD (108). These studies suggest that significant involvement of CCL/CCR pathways in macrophage recruitment and that the inhibition of these pathways showed potential therapeutic effects. However, monocyte recruitment to the liver when injury ensues does not only have negative consequences, but can also have positive implications, as shown in a study in which CSF1-Fc fragment promoted KC proliferation and monocyte infiltration and differentiation, restored innate immunity (92). However, as described previously, monocytes can differentiate into a plethora of phenotypes with discrete functions depending on the microenvironmental cues. They can also differentiate into a phenotype that is involved in the restoration of organ damage depending on the liver disease and stage of the disease.

## Conclusion and Future Prospective

Research about the insights in the initiation and progression of liver diseases has given the opportunity to develop novel therapeutic targeting strategies for the treatment of different liver diseases. The liver consists of a heterogenic population of hepatic macrophages, called KCs and MoMFs. Multiple studies have shown that hepatic macrophages play a pivotal role in liver homeostasis and liver diseases. Hepatic macrophages have central functions in the progression and regression of liver inflammation, ALF, liver fibrosis, NAFLD, ALD, viral hepatitis, and HCC. Different approaches have been developed to target hepatic macrophages to treat these different liver diseases. Due to the rapid advancement in nanomedicine attributable to its versatile application from drug delivery to diagnosis and imaging, few nanotechnology-enabled therapeutic modalities such as liposomes and polymeric micelles have been successfully approved for cancer treatment while others are under clinical investigation. Liposome and micelles based nanomedicines cause low toxicity and have a cost-efficient production. These are recent advantages that could help in the clinical translation of these nanomedicines. However, most of them have not been extensively tested yet in context to liver (and macrophage) targeting. Another promising nanocarrier could be solid lipid nanoparticles (SLN), which are based on solid components and are stable at room- and body temperature, resulting in a prolonged drug release. SLN consist of a lipid core that can be functionalized and stabilized with polymers to reduce non-specific cellular uptake (115).

Targeting therapies based on reducing the activation of KCs have been investigated. These therapies are mostly based on inhibiting intracellular inflammatory signaling pathways. KCs activation could also be dampened by restoring the normal gut microbiome, with probiotics, antibiotics, fecal microbiota transfer and sequestration of bile acids (12). In another study, cadherin-11 (CDH11) was increased during liver fibrosis suggesting this protein as an important regulator during liver fibrosis (117). The expression of CDH11 in injured cells, such as HSCs and macrophages, has been shown to regulate myofibroblasts activation and ECM production during the development of fibrosis. Therefore, CDH11 could be a potential therapeutic target of macrophages for the treatment of liver fibrosis. Furthermore, therapies have been focused on reducing monocyte recruitment to the liver. These therapies are mostly based on interfering with the chemokine signaling for monocytes. But as mentioned earlier, MoMFs can be categorized as Ly-6C^high^ monocytes, that cause organ impairment, and Ly-6C^low^ monocytes, which are organ restorative (39). Another potential strategy is to restore normal liver function by switching Ly6C^high^ monocytes into Ly6C^low^ monocytes. With this switch the function of the MoMFs could be changed into restorative instead of destructive.

Furthermore, therapies have been focused on reducing monocyte recruitment to the liver. These therapies are mostly based on interfering with the chemokine signaling for monocytes. But as mentioned earlier, MoMFs can be categorized as Ly-6C^high^ monocytes, that cause organ impairment, and Ly-6C^low^ monocytes, which are organ restorative (39). Another potential strategy is to restore normal liver function by switching Ly-6C^high^ monocytes into Ly-6C^low^ monocytes. With this switch the function of the MoMFs could be changed into restorative instead of destructive.

The recent studies have unraveled the large spectrum of macrophage phenotypes suggesting the heterogeneity and immunomodulatory functions of macrophages in liver diseases. Inspired by the recent developments, the questions that remain unanswered are: what is the significance and function of different macrophage phenotypes, can we reprogram the selective phenotypic macrophages for disease resolution and, finally how can we induce disease regression without affecting functions of other macrophage phenotypes and other cell types thereby reducing adverse effects? Taken together, the understanding of macrophage heterogeneity and their role in liver diseases gives the opportunity to translate this knowledge into developing targeted therapies to treat these diseases in the clinic.

## Author Contributions

DH and RB drafted the manuscript and designed the figures. RB and RW made the final corrections. All authors corrected and approved the manuscript.

### Conflict of Interest

The authors declare that the research was conducted in the absence of any commercial or financial relationships that could be construed as a potential conflict of interest.
